# In Vivo Biosynthesis and Direct Incorporation of Noncanonical Amino Acids into Proteins

**DOI:** 10.1002/cbic.202500282

**Published:** 2025-09-19

**Authors:** Jan Hendrik Illies, Tim Moritz Weber, Ivana Drienovská

**Affiliations:** ^1^ Amsterdam Institute of Molecular and Life Sciences (AIMMS) Vrije Universiteit Amsterdam De Boelelaan 1105 1081HV Amsterdam The Netherlands; ^2^ Department of Chemistry and Pharmaceutical Sciences Vrije Universiteit Amsterdam De Boelelaan 1105 1081HV Amsterdam The Netherlands

**Keywords:** autonomous cells, biosynthesis, genetic code expansion, noncanonical amino acids, protein engineering

## Abstract

Autonomous cells are engineered biological systems capable of biosynthesising and directly incorporating noncanonical amino acids (ncAAs) into proteins. These systems have the potential to extend the applicability of the genetic code to enable large‐scale fermentative production of proteins carrying ncAAs. This work evaluates approaches for the generation of autonomous and semi‐autonomous cells. Semi‐autonomous cells rely on the external addition of a precursor, which is enzymatically converted in vivo to an ncAA that is directly incorporated. In contrast, autonomous cells have a metabolic system that produces and directly incorporates an ncAA in vivo. Through a critical evaluation of the state of the art, the reader is provided with an opinion on the future development of the field.

## Graphical abstract

This review evaluates engineered (semi)autonomous cell systems for the biosynthesis and incorporation of noncanonical amino acids (ncAAs) into proteins. While semi‐autonomous cells convert supplied precursors into ncAAs autonomous cells integrate biosynthetic pathways that produce these building blocks intracellularly. Such integrated approaches significantly reduce process costs, can increase protein yields, and overcome challenges such as the limited membrane permeability of ncAAs.

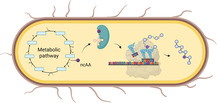

## 1 Introduction

Proteins, including enzymes used in the biotechnology industry, often need to meet a wide range of requirements, including excellent activity, selectivity, and stability.^[^ ^1^ ^]^ In many cases, directed evolution, rational design, or metagenomic and in silico screening are required to achieve these properties.^[^ ^2^ ^]^ However, the repertoire of functional groups implemented is limited by the 20 canonical amino acids and the two unusual amino acids pyrrolysine and selenocysteine.

Noncanonical amino acids (ncAAs) hold the potential to significantly expand the functional group diversity in proteins, including enzymes, surpassing the performance of natural enzymes. This potential opens up a world of possibilities for researchers in biotechnology and biochemistry, inspiring them to push the boundaries of their research.^[^ ^3^ ^]^ Expanded and new functionalities include electron donating or withdrawing groups,^[^ ^4^ ^]^ azides or alkynes for biorthogonal labeling,^[^ ^5^ ^]^ but also properties such as modulation of pK_a_ values,^[4b]^ stability,^[^ ^6^ ^]^ and hydrophobicity.^[^ ^7^ ^]^ This expanded set can also be used to drive progress in biocatalysis and catalyze new‐to‐nature reactions.^[^ ^8^ ^]^

Two approaches are commonly used to incorporate ncAAs in vivo: selective pressure incorporation (SPI), also known as residue‐specific incorporation, and stop codon suppression (SCS), also known as site‐specific incorporation. In the SPI method, auxotrophic strains are generally used, and a structural analog of a canonical amino acid is provided. This ncAA is then incorporated into the protein by the strain's own translation machinery.^[^ ^3^, ^9^ ^]^ On the other hand, the SCS method allows the site‐specific incorporation of ncAAs into proteins using an orthogonal translation system (OTS) consisting of an aminoacyl‐tRNA synthetase that loads the ncAA onto an orthogonal tRNA carrying a CUA anticodon. This OTS, which is also often referred to as an orthogonal pair (abbreviated: o‐pair), must be specific for the ncAA and should neither cross‐react with the host tRNAs nor the canonical amino acids. The site of ncAA incorporation in the target protein is typically defined by an in‐frame amber stop codon (TAG), although alternative and expanded codons can also be employed. The orthogonal tRNA recognizes the TAG codon via its complementary anticodon, enabling precise incorporation of the ncAA into the protein sequence leading to a genetic code expansion. For detailed explanations, please refer to one of the many good reviews that have covered this topic in detail.[^8^, ^10^]

However, both incorporation systems usually require an external supply of ncAAs. Due to the costs and level of ncAAs required, it is necessary that the benefits of ncAA incorporation into proteins outweigh the expenses to make large‐scale production of proteins with incorporated ncAAs (alloproteins) worthwhile. One strategy to reduce the high cost of using ncAAs is to biosynthesize them directly in the host organism, where they are incorporated into the protein of interest. This can be achieved either by providing a precursor that is converted to an ncAA and incorporated directly (semi‐autonomous cells), or by developing a biosynthetic pathway in the host that leads to an ncAA for direct incorporation (autonomous cells).

The economic feasibility of the (semi)autonomous cell approach, as demonstrated by the significant cost reduction of nearly 30‐fold,^[^ ^11^ ^]^ underlines the potential impact of this method to produce alloproteins.

This review considers literature up to March 2025. While some more recent works are cited to acknowledge ongoing developments, they are not reviewed in detail.^[^ ^12^ ^]^

## 2 (Semi)Autonomous Cells: Combining Biosynthesis and Incorporation of ncAAs

### 2.1 Semi‐autonomous Cells: Biosynthesis of ncAAs via a Precursor and Incorporation into Proteins

**Figure 1:**
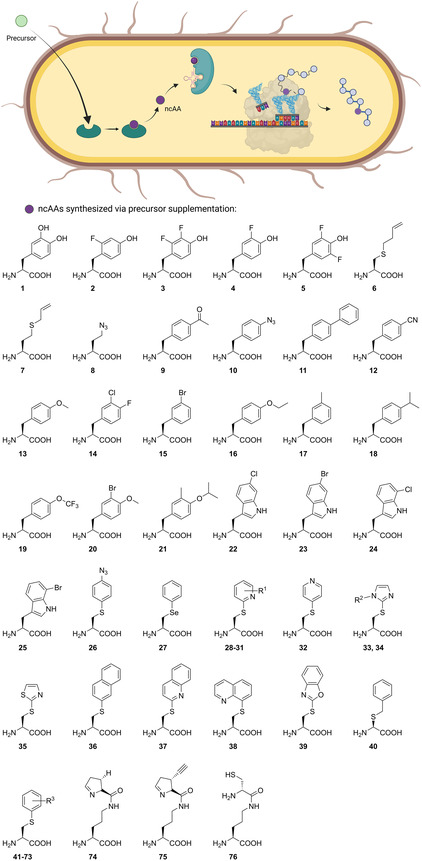
Schematic overview of a semi‐autonomous cell system including the supplementation of an ncAA precursor, conversion to the ncAA, and incorporation into a protein via an endogenous aminoacyl‐tRNA synthetase and tRNA in case of SPI or an OTS in case of SCS. The ncAAs derived in semi‐autonomous cell approaches are further specified. R^1^: Br, Cl, H, CF_3_; R^2^: H, Me; R^3^: Ac, AcNH, NH_2_, N_3_, Br, COOH, CH_2_COOH, Cl, CN, F, H, OH, *i*‐Pr, OMe, Me, NO_2_, CF_3_. The figure was partially created in https://BioRender.com.

A large number of biosynthetically produced and directly incorporated ncAAs are based on the addition of a precursor that is enzymatically converted to the ncAA of interest, leading to so called semi‐autonomous cells (**Figure** **1**). This section describes in detail the approaches chosen to engineer these semi‐autonomous cells capable of producing alloproteins.

In an approach to create semi‐autonomous cells, Kim et al. biosynthesized and directly incorporated l‐3,4‐dihydroxy‐phenylalanine (DOPA, **1**),^[^ ^11^ ^]^ utilizing emerald green fluorescent protein (GFP) as a model protein. To this end, several enzymes were evaluated for their suitability in DOPA biosynthesis. Tyrosine phenol lyase (TPL) was chosen because it can catalyze the breakdown of tyrosine to phenol, ammonia, and pyruvate, as well as the reverse reaction. This is important because high tyrosine levels can lead to misincorporation, which makes TPL's ability to regulate tyrosine levels particularly valuable. In addition, TPL can synthesize DOPA when catechol is used as the substrate instead of phenol. The OTS of *Methanocaldococcus jannaschii* tyrosyl‐tRNA synthetase and tRNA (*Mj*TyrRS) was used to incorporate DOPA into emerald GFP. This system had previously been engineered to incorporate DOPA,^[^ ^13^ ^]^ but further optimization was required to reduce misincorporation with Tyr and increase the efficiency of the OTS. After optimizing the biosynthesis and incorporation system independently, the two approaches were combined. The efficiency of feeding DOPA directly with feeding catechol as a precursor for biosynthesis was compared. The highest protein yield, with 98% DOPA incorporation, was achieved by supplying 10 mM catechol, producing 5.1 mg L^−1^ protein. In contrast, feeding 3 mM DOPA yielded only 1.8 mg L^−1^ protein. Therefore, the biosynthesis approach yielded almost three times as much protein as direct feeding of DOPA. A cost comparison further highlighted the advantages of the biosynthetic method. Producing 1 mg of protein using DOPA biosynthesis from catechol costs $0.05, whereas feeding DOPA costs $1.47 per mg of protein. This significant cost reduction underlines the economic feasibility of the biosynthesis approach to produce ncAA‐modified proteins. The utility of this system was further demonstrated by labeling maltose binding protein with a fluorophore via strain‐promoted oxidation‐controlled cyclooctyne‐1,2‐quinone cyclooxidation (SPOCQ). Therefore, biosynthesized DOPA was incorporated into maltose binding protein, subsequently oxidized, and subjected to SPOCQ. In this way, the protein was selectively labeled with a fluorophore, demonstrating the versatility of site‐specific DOPA incorporation for protein labeling and post‐translational functionalization. In another study using TPL, Won et al.^[^ ^14^ ^]^ biosynthesized a variety of fluorophenol ncAAs to improve the thermostability of industrially relevant enzymes by incorporating these ncAAs via SPI. They used a Tyr auxotrophic *Escherichia coli* strain harboring two plasmids: one with TPL under a constitutive promoter and another with a model protein. Initial optimization efforts were guided by the established DOPA biosynthesis system. The researchers found that supplementation with 20 mM catechol yielded the highest amounts of DOPA and observed that native pyruvate levels were sufficient for effective ncAA biosynthesis. However, cytotoxicity displayed by the fluorophenol substrate limited the viable concentration of the fluorinated precursor to a maximum of 3 mM. In addition, the feeding of tri‐ and tetra‐substituted fluorotyrosines as substrates for the TPL‐based ncAA biosynthesis resulted in lower protein yields, leading the researchers to focus on mono‐ and disubstituted fluorotyrosines, specifically 2‐fluorotyrosine (2‐FY, **2**), 2,3‐difluorotyrosine (2,3‐FY, **3**), 3‐fluorotyrosine (3‐FY, **4**), and 3,5‐difluorotyrosine (3,5‐FY, **5**). Combining biosynthesis and incorporation into a Tyr auxotrophic *E. coli* strain resulted in the replacement of all Tyr residues of GFP by their fluorinated ncAA analogs **2–4**, except for 3,5‐FY **5**. Furthermore, incorporating 2‐FY **2** into ω‐transaminase and alanine dehydrogenase increased the melting temperature by ≈4 °C and residual activity after incubation at elevated temperatures. This underlined that fluorinated ncAAs can improve thermal stability for enzymes of industrial relevance.

Utilizing two pyridoxal‐phosphate (PLP)‐dependent lyases called *O*‐acetyl‐serine sulfhydrylase isoenzymes (OASS), which are native to *E. coli*, Budisa and colleagues^[^ ^15^ ^]^ biosynthesized and incorporated *S*‐allyl‐l‐cysteine (SAC, **6**). Therefore, the small molecule precursor allyl mercaptan was supplemented, and native levels of *O*‐acetylserine were used to create semi‐autonomous *E. coli* cells. After developing an OTS derived from *Methanosarcina mazei* pyrrolysine aminoacyl‐tRNA synthetase and tRNA (*Mm*PylRS) to incorporate SAC, the researchers focused on establishing a single‐step biosynthesis. They first studied the isoenzymes OASS‐A and OASS‐B in vitro, with OASS‐B showing higher promiscuity toward allyl mercaptan. The final route of biosynthesis and incorporation was the cultivation of *E. coli* BL21(DE3) harboring a reporter plasmid containing enhanced GFP (EGFP) and another plasmid containing the modified *Mm*PylRS/tRNA OTS. After pregrowth, the precursor allyl mercaptan (2 mM) was added, and EGFP_150TAG expression was induced, resulting in EGFP_150SAC. Native levels of OASS‐B were used for biosynthesis, as the overexpression did not result in higher EGFP_150SAC yields. The study also described SAC as suitable for bioconjugation. Incorporation of SAC into a cysteine‐free superfolder GFP (sfGFP) facilitated the attachment of the protein to a thiol‐functionalized hydrogel via a thiol‐ene reaction, demonstrating a versatile approach that could be applied in industrial biocatalysis. Further, the use of cysteine‐free sfGFP to introduce a protected cysteine into a protein has been illustrated. After incorporating SAC, reduction to Cys via Pd^0^ was demonstrated. In a later study, Budisa and colleagues^[^ ^16^ ^]^ synthesized the homo analog of SAC—*S*‐allyl‐l‐homocysteine (SAHC, **7**)—and incorporated it into GFP. While the structures of SAC and SAHC are similar, their precursors and the pathways leading to them are different. Unlike SAC, SAHC is a product of the Met metabolism. In addition, SAHC can be incorporated via the endogenous bacterial MetRS, which was not the case for SAC. Therefore, when considering the biosynthesis of SAHC, the *Corynebacterium glutamicum* pathway was of interest because homoserine acetyltransferase (*Cg*HSAT) natively converts l‐homoserine (derived from the tricarboxylic acid cycle) to *O*‐acetyl‐l‐homoserine (OaHs). OaHs can be converted to SAHC via PLP‐dependent *C. glutamicum* *O*‐acetyl homoserine sulfhydrylase (*Cg*OAHSS), with allyl mercaptan acting as a nucleophile. Taking advantage of this pathway and the possibility of linking SAHC biosynthesis to incorporation via MetRS and SPI, a Met‐auxotrophic *E. coli* B834(DE3) strain was constructed, carrying a plasmid with the genes *metX* and *metY* encoding *Cg*HSAT and *Cg*OAHSS, respectively. Next to allyl mercaptan, pantothenic acid was added to enhance the biosynthesis of the precursor l‐homoserine. Although incorporation of SAHC into GFP was successfully shown, the low levels of incorporation with multiple Met codons in the sequence led to the assumption that the levels of biosynthetically produced SAHC were too low for full incorporation or that the remaining Met levels were too high. Nonetheless, several applications of SAHC biosynthesis and incorporation were highlighted in this work: photoinduced orthogonal thiol‐ene coupling, immobilization on a hydrogel, and reduction via Pd^0^ for demasking of l‐homocysteine.

**Scheme 1:**
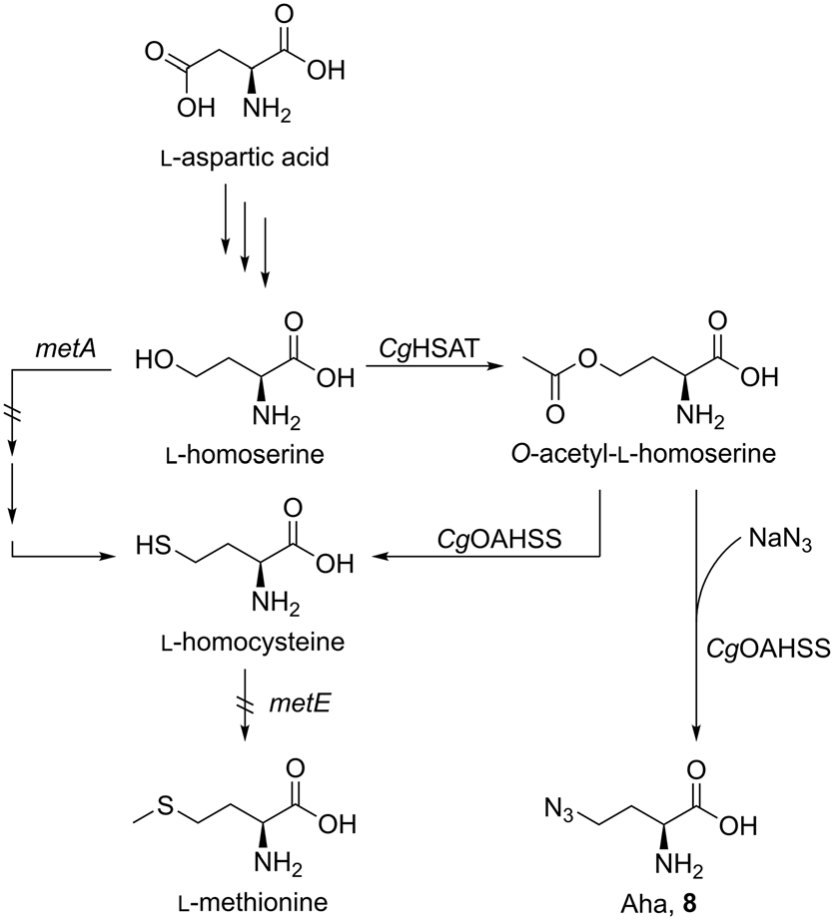
Engineered Met metabolism in *E. coli*. The genes *metA* and *metE* were knocked out to minimize Met levels to avoid Met misincorporation. The recombinantly expressed enzymes *Cg*HSAT and *Cg*OAHSS from *C. glutamicum* lead, under supplementation of NaN_3_, to the conversion of l‐homoserine to Aha **8**.

Working on the biosynthesis and direct incorporation of l‐azidohomoalanine (Aha, **8**), Budisa, di Salvo, and colleagues^[^ ^17^ ^]^ made use of OAHSS from *C. glutamicum*. Two plasmids were introduced into the methionine auxotrophic strain *E. coli* B834: one carrying *metY* encoding *Cg*OAHSS and another plasmid encoding the cysteine‐free ψB* protein commonly used for folding studies. The ψB* variant was designed to contain either a methionine ATG codon at the N‐terminal position or at an internal position, to be replaced by the ncAA. A two‐step process was developed to achieve biosynthesis and incorporation of the ncAA. In the first step, cells were grown in a minimal medium supplemented with Met, during which recombinant expression of *Cg*OAHSS was induced. In the second step, *O*‐acetyl‐l‐homoserine and NaN_3_ were added to a final concentration of 1 mM and ψB* expression was induced. The incorporation of Aha into ψB* was successfully demonstrated both at the N‐terminal position and at the internal position. Purified ψB*_1Aha was then conjugated with dansylpropargylamine via copper(I)‐catalyzed azide‐alkyne cycloaddition (CuAAC). This approach effectively demonstrated that the biosynthesis and direct incorporation of an ncAA enables the site‐specific labeling of proteins with fluorophores. Building on their previous work^[^ ^17^ ^]^ of Aha biosynthesis with simultaneous incorporation, Budisa, di Salvo, and colleagues^[^ ^18^ ^]^ established an Aha biosynthetic pathway that eliminated the need for costly supplementation of the l‐homoserine precursor. A streamlined metabolic flux required engineering of the Met metabolism to reduce Met levels within the cells further and increase the accumulation of the metabolic precursor l‐homoserine (**Scheme** **1**).

Using a strain with knocked‐out *metA* allows the accumulation of higher levels of the metabolic precursor l‐homoserine. A strain with a knocked‐out *metA* gene was selected, which encodes the enzyme homoserine succinyltransferase, catalyzing the reaction of l‐homoserine to *O*‐succinyl‐l‐homoserine. Furthermore, the *S*‐methyltransferase *metE* was knocked out to prevent the undesired formation of Met from l‐homocysteine. By minimizing Met levels, less misincorporation of Met instead of Aha should occur. Adaptive laboratory evolution with respect to growth conditions led to the auxotrophic *E. coli* strain MDS15A. This strain was transformed with a plasmid containing the genes encoding for different model proteins and a plasmid with genes coding for *Cg*HSAT and *Cg*OAHSS. The biosynthesis was also optimized concerning the supplementation of NaN_3_ and pantothenic acid to increase the amount of Aha‐labeled target protein. Incorporating Aha into the protein of interest was then carried out similarly to the abovementioned approach.^[^ ^17^ ^]^ Although this approach improved the effective biosynthesis and incorporation of Aha, not all target proteins tested were completely homogeneous. However, this approach allowed the metabolic accumulation of the metabolic precursor l‐homoserine, eliminating the need for its costly supplementation.

While Budisa, di Salvo, and colleagues focused on optimizing the availability of metabolic precursors, other approaches have explored the enzymatic synthesis of ncAAs using promiscuous aminotransferases. One such strategy, developed by Jung et al.^[^ ^19^ ^]^ utilized the glutamine:phenylpyruvate aminotransferase (GlnAT) from *Thermus thermophilus* HB8 to synthesize ncAAs by supplementing the corresponding keto acids. When testing in vitro with purified enzyme, numerous ncAAs were synthesized with 50–82% activity of the natural substrate, such as *p*‐acetyl‐l‐phenylalanine (*p*AcF, **9**), *p*‐azido‐l‐phenylalanine (*p*AzF, **10**), *p*‐phenyl‐l‐phenylalanine (*p*PhF, **11**), and *p*‐cyano‐l‐phenylalanine (*p*CNF, **12**). Different predeveloped OTSs derived from *M. jannaschii* were used for incorporation.^[^ ^20^ ^]^ To combine biosynthesis and incorporation, a two‐plasmid system was used in *E. coli* BL21(DE3); one plasmid carried the genes expressing the aminotransferase GlnAT and the GFP_151TAG reporter protein, and the other carried the OTS. Higher GFP yields were obtained by supplementing 2 mM alpha‐keto acids compared to supplementing 1 mM ncAA in the cases of *p*AcF (23 mg L^−1^ vs 13 mg L^−1^) and *p*AzF (22.4 mg L^−1^ vs 17.2 mg L^−1^). The levels of GFP with incorporated biosynthetic *p*PhF and *p*CNF were slightly lower in contrast to supplemented ncAA (1 mM). In another study using GlnAT, Liu et al.^[^ ^21^ ^]^ used the native levels of *E. coli* transferases (GlnAT/AspAT) and supplemented keto acid precursors to biosynthesize and incorporate a variety of ncAAs into EGFP_149TAG via SCS. A two‐plasmid system, one containing EGFP_149TAG and the other a mutant of the *M. mazei* derived orthogonal pyrrolysine pair called pEvolve‐*methanosarcina mazei* aminoacyl‐tRNA synthetase (MmRS), was constructed. First, the authors tested the interaction between *p*‐methoxyphenylpyruvic acid (*p*MeOPPA) and GlnAT in terms of the conversion of *p*MeOPPA into l‐4‐methoxyphenylalanine (OMeY, **13**) and its subsequent incorporation into EGFP in different *E. coli* strains, namely C321.A.exp, C321.A.expΔPBAD, DH10B, Top10F, and BL21AI. The latter two did not undergo biotransformation and incorporation into EGFP, while DH10B showed the strongest fluorescence signal, suggesting efficient biotransformation of *p*MeOPPA to OMeY and subsequent incorporation into EGFP. In a screening using different variants of phenylpyruvic acid with differing functional groups in the *para*‐ or *meta*‐position, the biosynthesis and incorporation of the corresponding ncAAs **14–21** (Figure 1) into EGFP was achieved. This approach, using native transferase levels and expression of the OTS and reporter protein, resulted in the incorporation of 9 out of 13 keto acid precursors evaluated. In another approach using a transferase for ncAA biosynthesis, Xiao and colleagues^[^ ^22^ ^]^ biosynthesized and incorporated four halogenated Trp analogs. For the incorporation, different OTSs were validated. The best incorporation efficiency was achieved using two variants derived from the chimeric human mitochondrial phenylalanyl‐tRNA synthetase and the corresponding tRNA. For the incorporation of 6‐chloro‐tryptophan (6ClW, **22**) and 6‐bromo‐tryptophan (6BrW, **23**), the chPheRS‐3 and chimeric phenylalanyl‐tRNA (3C11‐chPheT) OTS^[^ ^23^ ^]^ were used, and the chPheRS‐4/3C11‐chPheT OTS^[^ ^23^ ^]^ for the incorporation of 7‐chloro‐tryptophan (7ClW, **24**) and 7‐bromo‐tryptophan (7BrW, **25**). Four different flavin‐dependent Trp halogenases were evaluated to establish the biosynthesis. To provide an efficient electron transport chain and regeneration system for the flavin cofactor FADH_2_, the Trp halogenases were translationally fused to the *E. coli* flavin reductase. The halogenation efficiency was evaluated by incorporation into sfGFP, and the cheap halide salts NaCl and NaBr were added as bromide and chloride sources, respectively. For halogenation of the tryptophan C‐6 position and subsequent incorporation, usage of the SttH halogenase resulted in the highest fluorescence. In contrast, RebH halogenase provided the highest fluorescence for halogenation and incorporation in the tryptophan C‐7 position. Further, the versatility of the system was demonstrated via the incorporation of the halogenated Trp analogs into nanoluciferase and the single‐chain variable fragment of antihuman epidermal growth factor 2 antibody (anti‐HER2).

**Scheme 2:**
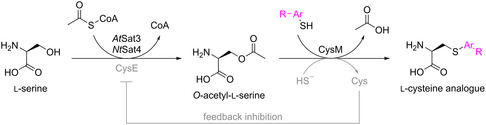
Biosynthetic pathway toward l‐cysteine analogs. The two cysteine‐insensitive *O*‐acetyltransferases *At*Sat3 and *Nt*Sat4, as well as native CysM, were overexpressed. Aromatic thiols were supplemented as ncAA precursors. Purple highlighted Ar‐R corresponds to ncAAs **26–73** in Figure 1. The scheme was adapted from Liu et al. with permission from ref. [24] (Copyright Wiley, 2021).

Wang et al.^[^ ^24^ ^]^ demonstrated the biosynthesis and incorporation of nearly 50 ncAAs (**26–73**) into a protein by supplementing a remarkable diversity of precursors to the same system (**Scheme** **2**).

The study focused on the cysteine biosynthetic pathway, where the PLP‐dependent enzyme CysM exhibits substrate promiscuity, enabling substitution of its natural substrate, α‐aminoacrylate intermediate, with various nucleophiles. In an in vitro assay using purified CysM, the rapid conversion of sodium benzenethiolate to *S*‐phenyl‐l‐cysteine was used to demonstrate the promiscuity of CysM for thiol nucleophiles. To further optimize the metabolic flux in the proposed ncAA biosynthesis, the authors aimed to increase the availability of the *O*‐acetylated metabolic precursor. Consequently, a strategy was needed to circumvent the tight regulation of native CysE. Therefore, the *O*‐acetyl‐*S*‐transferases *Nt*Sat4 from *Nicotiana tabacum* ^[^ ^25^ ^]^ and *At*Sat3 from *Arabidopsis thaliana*,^[^ ^26^ ^]^ which convert l‐serine to *O*‐acetyl‐l‐serine and are known to be insensitive to feedback regulation and transform l‐serine to *O*‐acetyl‐l‐serine, were overexpressed in the bacterial host. Incorporation was first tested using *S*‐phenyl‐l‐cysteine and the previously developed *M. jannaschii* OTS.^[^ ^27^ ^]^ Combining biotransformation of thiol nucleophiles and direct incorporation of the novel ncAAs, a three‐plasmid system was developed for biosynthesis and incorporation into *E. coli* BL21(DE3). Using this system, the authors were able to achieve up to 45 mg L^−1^ purified sfGFP by supplementing 1 mM sodium benzenethiolate. Further, this approach was applied to 49 other aromatic thiol precursors to synthetize and directly incorporate the corresponding ncAA. In this way, proteins that can be used for various biotechnological applications, such as site‐specific labeling, utilization as ^19^F‐NMR probe, and for site‐specific protein functionalization, were expressed. The authors were particularly interested in *S*‐(4‐azidophenyl)‐l‐cysteine (*p*AzPhC, **26**) because of its potential as a bioorthogonal handle due to the presence of the azide‐group in the side chain. By optimizing four positions of the *Mj*TyrRS OTS, the yield of purified protein was increased from 39 to 70 mg L^−1^. To demonstrate the applicability of this approach, they biosynthesized *p*AzPhC and incorporated it into the antigen‐binding fragment of the anti‐HER2 antibody by adding 1 mM of the precursor. This resulted in 1.5 mg L^−1^ protein, which is remarkable compared to 2 mg L^−1^ of wild‐type protein. Treatment with the click‐reactive dibenzocyclooctyne probe (DBCO)‐mPEG20K gave a similar efficiency in strain‐promoted cross‐linking to *p*AzF, the standard ncAA used in click chemistry.

**Scheme 3:**
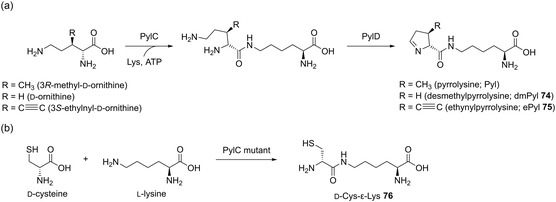
Biosynthetic pathway toward pyrrolysine derivates. a) Transformation of 3*R*‐methyl‐d‐ornithine, d‐ornithine, and 3*S*‐ethynyl‐d‐ornithine to pyrrolysine, dmPyl **74**, and ePyl **75**, respectively, via the enzymes PylC and PylD. b) Biotransformation of d‐cysteine and l‐lysine to d‐Cys‐ε‐Lys **76** via a PylC mutant.

In an approach using the enzymes of pyrrolysine synthesis and the corresponding OTS, Ou et al.^[^ ^28^ ^]^ biosynthesized desmethylpyrrolysine (dmPyl, **74**) and incorporated it into a variety of proteins using *E. coli* and HEK cells as host organisms. To generate semi‐autonomous cells, a two‐plasmid system was used. While one plasmid contained the pyrrolysine biosynthesis enzymes PylC and PylD (**Scheme** **3**a), as well as the OTS, the second plasmid contained the protein of interest. Proteins of interest for the heterologous production in *E. coli* were the human fatty acid synthase, mouse tumor necrosis factor alpha, FK506 binding protein 1A, and mouse epidermal growth factor. For mammalian host cells, human retinal binding protein 4 or mouse erythropoietin was targeted instead. Either *E. coli* BL21(DE3) or HEK293F cells were chosen as the expression strain and fed 5 mM d‐ornithine as the precursor for dmPyl. The protein yields were 25–40% of the wild‐type protein in most cases. For HEK cells, 10–20% of the wild‐type expression level was achieved. For biorthogonal conjugation, the incorporated dmPyl was derivatized with 2‐aminobenzaldehyde or 2‐amino‐acetophenone. Lastly, more than 50 different dmPyl‐reactive reagents consisting of small molecules, PEG polymers, and fluorescent labels were conjugated with the alloproteins. Ehrlich et al.^[^ ^29^ ^]^ also exploited two enzymes involved in pyrrolysine biosynthesis. With this, they synthesized and directly incorporated dmPyl (**74**) and 3*S*‐ethynylpyrrolysine (ePyl, **75**). The biosynthetic precursors (d‐ornithine and 3*S*‐ethynyl‐d‐ornithine) were provided by a multistep organic synthesis. The enzymes PylC and PylD natively ligate 3*R*‐methyl‐d‐ornithine with Lys and subsequently oxidize the isopeptide to pyrrolysine (Scheme 3a). For biosynthesis and incorporation, three plasmids were used: one plasmid containing the genes corresponding to PylC and PylD, one plasmid containing a copy of the PylRS tRNA synthetase and three copies of the corresponding tRNA, and chloramphenicol acetyltransferase with a TAG stop codon for positive selection. The third plasmid contains the human carbonic anhydrase 2 (hCA) gene with an in‐frame TAG codon. The resulting yields of hCA_dmPyl and hCA_ePyl were 6 and 75 mg L^−1^ of isolated protein, respectively. In combination with growth studies, it was concluded that the efficiency of the biosynthesis was the reason for the different protein yields. To demonstrate the capabilities of the incorporated ncAA ePyl, two biorthogonal protein modifications were introduced. In one example, the terminal alkyne was reacted with 7‐hydroxycoumarin azide via CuAAC, generating a fluorescent probe. Second, the imine moiety of the pyrroline ring was reacted with 2‐aminobenzaldehyde to form a tertiary amine.

Using the two reactive groups of ePyl, namely alkyne and imine, enabled simultaneous alloprotein labeling by two different orthogonal reactions. In another approach using the pyrrolysine biosynthetic pathway, Tai et al.^[^ ^30^ ^]^ utilized a PylC mutant to synthesize d‐Cys‐ε‐Lys (**76**) and incorporate it directly into a therapeutic peptide for cyclization (Scheme 3b). First, an OTS for ncAA incorporation was sought, and a library of tRNA aminoacyl synthetase PylRS mutants was screened, resulting in PylRS^EVF^, which was used together with tRNA^M15^ for d‐Cys‐ε‐Lys initial incorporation into mCherry as a model protein. *E. coli* C321.ΔA.M9adapted^[^ ^31^ ^]^ was tested against *E. coli* BL21(DE3) to improve the incorporation efficiency further. This resulted in a 5.3‐fold improvement in mCherry fluorescence when using *E. coli* C321.ΔA.M9adapted. To further implement the biosynthesis of d‐Cys‐ε‐Lys, PylC, which naturally catalyzes the coupling of Lys and 3‐methyl‐d‐ornithine, was targeted. This was to be re‐engineered to couple Lys and d‐Cys to form d‐Cys‐ε‐Lys. A PylC library was generated, and the resulting improved mutant was called PylC^NPSV^. The readthrough efficiency was assessed by sodium dodecyl sulfate‐polyacrylamide gel electrophoresis and indicated that supplementation with 5 mM d‐Cys gave an efficiency similar to that of exogenous feeding with 4 mM d‐Cys‐ε‐Lys. To demonstrate an application for the utility of semi‐autonomous biosynthesis and incorporation of d‐Cys‐ε‐Lys in peptide cyclization, the authors incorporated the isopeptide ncAA at the N‐terminus of a 23‐mer peptide, called p16p, which is derived from the binding sequence of the tumor suppressor protein P16. The P16 protein acts as a tumor suppressor because it inhibits cyclin‐dependent kinase (CDK) 4/6, preventing retinoblastoma phosphorylation. p16p was cyclized between the introduced ncAA d‐Cys‐ε‐Lys and the C‐terminal thioester within 3 h. The cyclization resulted in a higher binding affinity to CDK and a stronger inhibition of retinoblastoma phosphorylation.

### 2.2 Autonomous Cells: Biosynthesis of ncAAs via a Metabolic Pathway and Incorporation into Proteins

**Figure 2:**
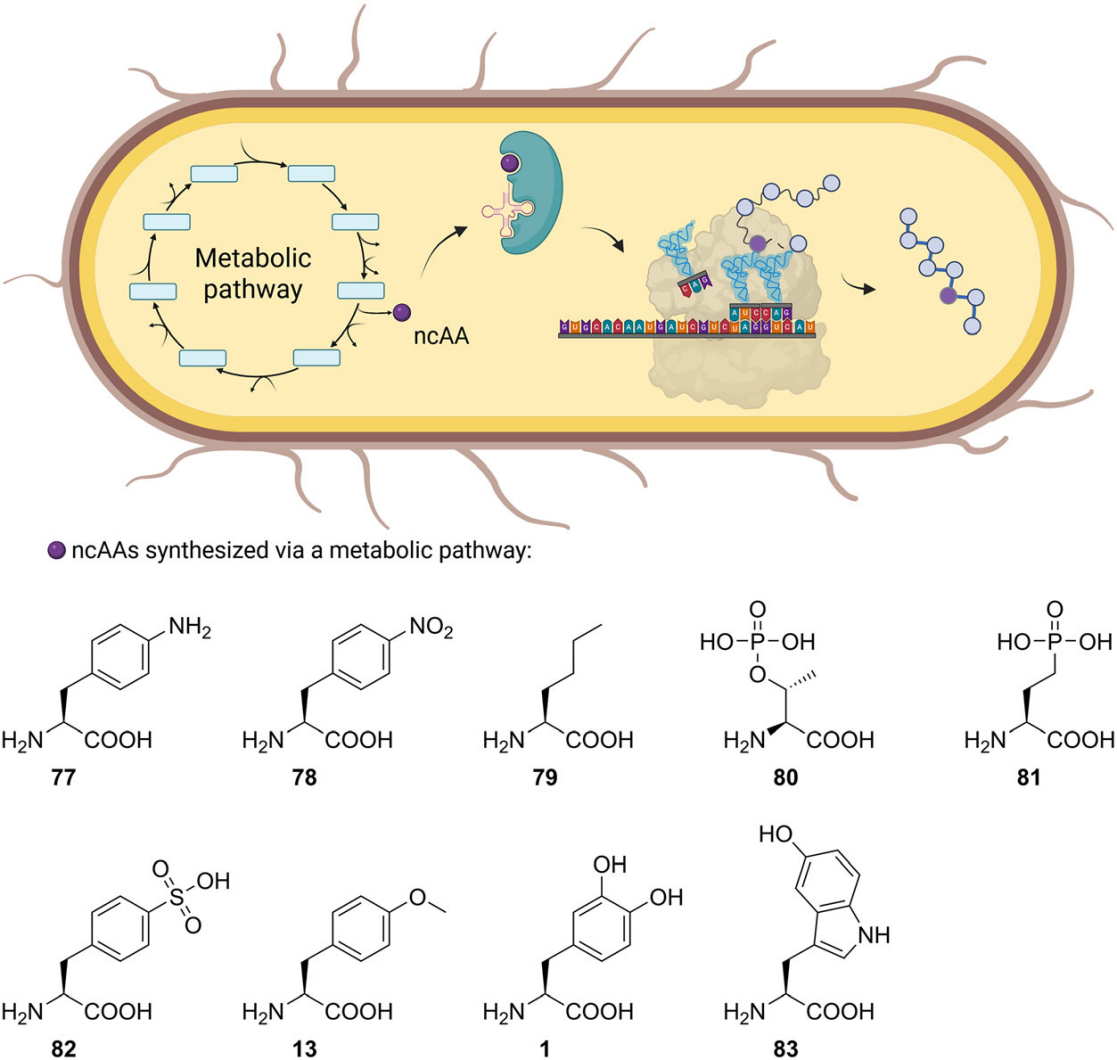
Schematic overview of an autonomous cell system. The conversion via a metabolic pathway to the ncAA and incorporation into a protein via an endogenous aminoacyl‐tRNA synthetase and tRNA in case of SPI or an OTS in case of SCS are illustrated. The ncAAs used in autonomous cell approaches are further detailed. The figure was partially created in https://BioRender.com.

This section outlines approaches focused on engineering specific metabolic pathways to biosynthesize ncAAs, which are then incorporated into proteins. This strategy enables the creation of truly autonomous cells without the need for additional precursor supplementation. **Figure** **2** summarizes the ncAAs incorporated within this type of autonomous cells.

**Scheme 4:**
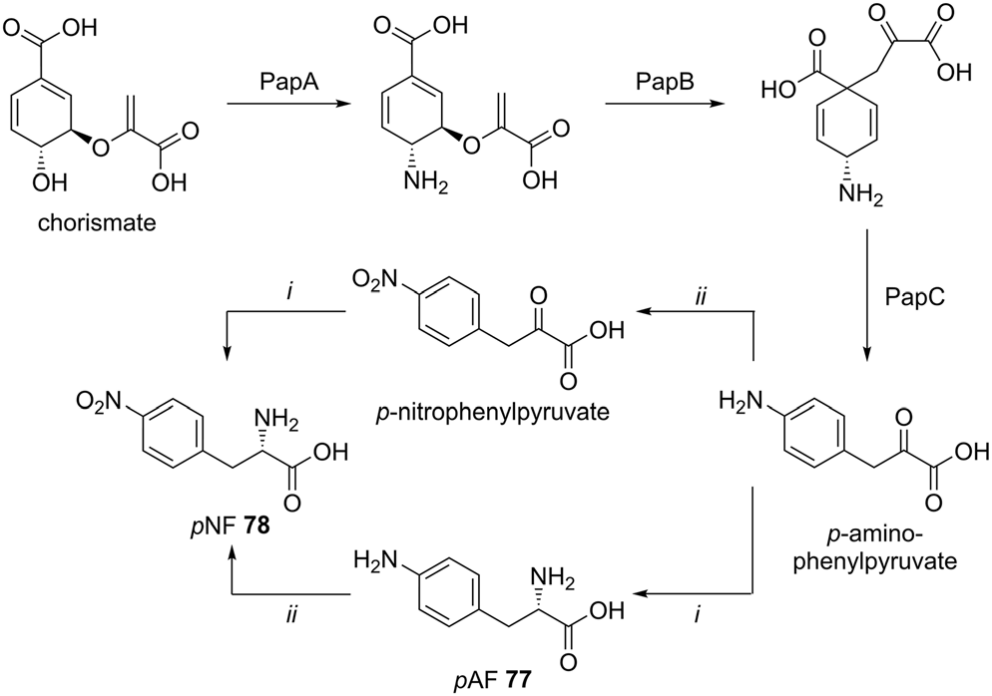
Biosynthetic pathway from chorismate to *p*AF **77** and *p*NF **78**. The genes coding for the enzymes PapA, PapB, and PapC were derived from *S. venezuelae* and catalyze the reactions from chorismate to *p*‐aminophenylpyruvate. In the approaches of the Schultz^[^ ^13^ ^]^ and Xiao^[^ ^33^ ^]^ groups, an endogenous *E. coli* aminotransferase i) was used, leading to *p*AF **77**. The approach of Butler et al.^[^ ^34^ ^]^ built up on this and used ObiL or NO16 ii) for the transformation to *p*NF **78**.

Schultz and coworkers^[^ ^32^ ^]^ were the first to produce an autonomous bacterium encoding 21 amino acids with an extended genetic code. For this, they developed an *M. jannaschii*‐derived system capable of incorporating the Tyr analog *p*‐aminophenylalanine (*p*AF, **77**) at a level of 1 mM by mutating five different sites on the tyrosine tRNA aminoacyl synthetase TyrRS. This OTS was called *p*AFRS. In *Streptomyces venezuelae*, *p*AF is a natural metabolic intermediate of the chloramphenicol biosynthesis, and the corresponding genes have been identified within the *pap* locus. To implement the *p*AF biosynthesis in *E. coli*, the researchers placed the genes corresponding to the PapABC enzymes either under an inducible lac promoter or the constitutive *lpp* promoter. The final conversion of the keto acid to *p*AF was thought to be conducted by one of *E. coli*'s promiscuous tyrosine aminotransferases (*ilvE*, *tyrB*, or *aspS*) (**Scheme** **4**). The combination of biosynthesis and incorporation into myoglobin yielded 1.8 mg L^−1^ protein when the biosynthetic genes were under the control of the constitutive *lpp* promoter. The supplementation of 1 mM *p*AF led to 3.0 mg L^−1^ myoglobin. This early approach demonstrated levels of ≈50% alloprotein yield via the artificial biosynthetic pathway compared to supplementation. Using the same enzymatic Pap pathway and OTS,^[^ ^32^ ^]^ Xiao and colleagues^[^ ^33^ ^]^ demonstrated the use of *p*AF alloproteins for biorthogonal ligation. To optimize the incorporation of *p*AF, *p*AFRS was cloned into the pUltra plasmid, in which *p*AFRS is controlled by the *proK* promoter and *p*AFRS is lacI‐driven. This setup resulted in a twofold increase in expression of *p*AF alloprotein compared to a pDule plasmid with an analogous OTS arrangement. For the cascade enzymes, PapABC from *S. venezuelae* were used under a *lpp* promoter. Using a three‐plasmid system for the biosynthesis and incorporation of *p*AF into the sfGFP reporter protein resulted in a final protein yield of 1 mg L^−1^, while exogenous feeding of 1 mM *p*AF resulted in 4.5 mg L^−1^ sfGFP. To further demonstrate an application of industrial relevance, *p*AF was incorporated into the HER2‐targeting anti‐HER2 antibody. Bioorthogonal oxidative coupling between the anti‐HER2_*p*AF antibody and functionalized Rhodamine B, which is a fluorescent dye, was used for the coupling reaction. This demonstrated that *p*AF alloproteins can be used to generate bioimaging tools. By combining the *p*AF biosynthetic pathway used in the previous approaches^[^ ^32^, ^33^ ^]^ with a non‐heme diiron *N*‐monooxygenase, Butler et al.^[^ ^34^ ^]^ biosynthesised and incorporated *p*‐nitrophenylalanine (*p*NF, **78**) into a reporter protein (Scheme 4). To create a route for the *p*NF synthesis, the ObiL *N*‐oxygenase was considered first due to its known ability to reduce *p*‐amino‐phenylpyruvate. However, in vitro tests confirmed only a 10% conversion of 1 mM of both tested substrates (*p*‐aminophenylpyruvate and *p*AF) after 3 h. A library of 21 sequences was screened to further improve the oxygenase activity, which was identified using a sequence similarity network. This led to the discovery of the previously uncharacterized enzyme NO16, which gave a twofold higher yield of *p*NF than ObiL. Careful selection of suitable expression hosts and optimization of expression conditions, regulation of feedback inhibition of *papABC* genes by coexpression of 3‐deoxy‐d‐arabino‐heptulosonate‐7‐phosphate synthase, and optimization of media finally led to a *p*NF titre of 820 μM. For incorporation of *p*NF, 20 different mutants of the *Mj*TyrRS aminoacyl tRNA synthetase were tested, finding that the previously reported TetRS‐C11^[^ ^35^ ^]^ resulted in the most efficient incorporation into a chimeric ubiquitin‐sfGFP target. Coupling the *p*NF biosynthesis and the ncAA incorporation resulted in 3.5 mg L^−1^ of isolated ubiquitin‐GFP, but additional peaks in liquid chromatography‐mass spectrometry (LC‐MS) revealed misincorporation with the precursor *p*AF (36%) and Tyr (17%). This demonstrated the utility of the biosynthesis but revealed that further development of the OTS is necessary.

In an approach focusing on the biosynthesis of norleucine (Nle, **79**), Anderhuber et al.^[^ ^36^ ^]^ demonstrated the feasibility of engineering autonomous cells which biosynthesize Nle and directly incorporate Nle via SPI. As targets of the incorporation, the model proteins old yellow enzyme (OYE)^[^ ^37^ ^]^ and the thermophilic lipase TTL were chosen. For the metabolic synthesis of Nle, the branched‐chain amino acid pathway was investigated. The concern was that the acetolactate synthase isoenzymes *ilvBN*, *ilbIH*, and *ilvGM* consume 2‐ketobutyrate, a crucial precursor for Nle biosynthesis. Aiming for ncAA incorporation via the aminoacyl‐tRNA synthetase MetRS, the Met auxotrophic strain *E. coli* B834(DE3) was chosen for the knockouts, which resulted in the quadruple auxotrophic *E. coli* BWEC14 strain, showing auxotrophy for the canonical amino acids Val, Ile, Leu, and Met. In comparison with strain B834(DE3), the streamlined metabolic flux in the engineered strain achieved four times higher Nle levels. Additionally, a plasmid with the leuA^fbr^BCD operon was introduced to increase Nle levels, as this operon is insensitive to feedback inhibition of free Leu. For the combination of biosynthesis and incorporation, Nle was first accumulated to levels of up to 5 g L^−1^ before the expression of OYE and TTL proteins was induced. The incorporation efficiency was reported to be higher than 90%. This global substitution with Nle is a potential way to prevent Met oxidation in proteins under oxidative conditions.

Incorporating charged ncAAs into proteins in vivo is challenging due to their limited ability to traverse bacterial membranes when provided externally. To address this, engineering autonomous cells synthesizing charged ncAAs intracellularly and directly incorporating them presents a promising strategy to enhance their incorporation efficiency. Zhang et al.^[^ ^38^ ^]^ established the biosynthesis of the charged amino acid *O*‐phospho‐l‐threonine (PThr, **80**) and its direct incorporation into several model proteins. The previously engineered phosphoseryl‐tRNA synthetase and tRNA OTS from *M. jannaschii* and *Methanococcus maripaludis* were used as the starting point for developing an OTS capable of incorporating PThr.^[^ ^39^ ^]^ First, the tRNA was optimized to avoid mis‐aminoacylation by endogenous aminoacyl‐tRNA synthetases, which led to the tRNA variant EF‐Sep. In the second step, a rapid parallel positive selection system was established. Finally, a variant (PThrRS‐tRNA^v2.0^_CUA_) was identified, carrying two mutations that facilitate selective incorporation of PThr. For biosynthesis, the threonine kinase from *Salmonella enterica* (PduX) was implemented into the *E. coli* host strain, allowing for the synthesis of PThr to levels of up to 1.7 mM. Combining biosynthesis and incorporation, the PThrRS‐tRNA^v2.0^_CUA_ pair, EF‐Sep tRNA, and kinase PduX were cloned into one plasmid and introduced alongside a second plasmid carrying the GFP_150TAG reporter. *E. coli* BL21(DE3)*ΔserC* was used as an expression strain, as the deletion of the phosphoserine aminotransferase *serC* was intended to minimize intracellular phosphoserine levels to reduce misincorporation of the cognate amino acid *O*‐phospho‐l‐serine (PSer). Combining the biosynthesis and incorporation systems resulted in 5 mg L^−1^ GFP. However, when this system was adapted to incorporate PThr into ubiquitin, a mixed incorporation of Thr and PThr was noticed. It was hypothesized that an *E. coli* phosphatase dephosphorylates incorporated PThr. Knocking out the putative phosphatase *ycdX* proved this hypothesis correct, as this modification of the host led to homogeneously phosphorylated ubiquitin. The authors also demonstrated the general applicability of these autonomous cells by incorporating PThr into CDK 2.

**Scheme 5:**
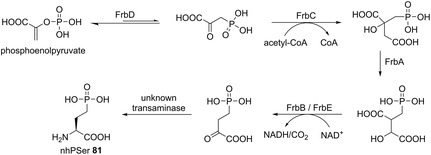
nhPSer biosynthetic pathway derived from *S.* *rubellomurinus*. FrbD is a phosphoenolpyruvate mutase that transforms the metabolic precursor phosphoenolpyruvate. The homocitrate synthase homolog FrbC tips the balance by irreversible acetyl‐CoA condensation. FrbA then acts as an aconitase, FrbB, and/or FrbE as an isocitrate dehydrogenase. An unknown *E. coli* transaminase finally leads to the formation of nhPSer **81**.

Phosphorylated amino acids undergo spontaneous hydrolysis in aqueous environments, as described for phosphoserine PSer and PThr. This undesired hydrolysis poses a great challenge when aiming for homogenous species of phosphorylated alloproteins. Zhu et al.^[^ ^40^ ^]^ biosynthesized and incorporated the nonhydrolysable PSer (nhPSer, **81**) derivative to mimic PSer but tackling the inherent instability. The previously developed system of nhPSer incorporation faced the challenge that charged amino acids do not permeate easily across cell membranes, resulting in low protein yields.^[^ ^39^ ^]^ Previous attempts also suggested that nhPSer could be produced by a pathway from the metabolic precursor phosphoenolpyruvate using the enzymes FrbD, FrbC, FrbA, and FrbB/FrbE derived from *Streptomyces rubellomurinus* ^[^ ^41^ ^]^ and an unknown transaminase from *E. coli* (**Scheme** **5**). To further improve this pathway, a combinatorial library of the individual enzymes under different T7 promoters was evaluated. A strong promoter for FrbC and FrbD was highly advantageous, tipping the balance toward nhPSer formation. For efficient biosynthesis and incorporation into sfGFP, the previously introduced *E. coli* BL21(DE3)*ΔserC* was used with three plasmids. One plasmid contained the OTS,^[^ ^39^ ^]^ and the overexpressed *serB* gene to hydrolyze PSer to avoid misincorporation. FrbABCDE, and the reporter plasmid carrying sfGFP_150TAG were located on the other two plasmids.

This resulted in 120 mg L^−1^ sfGFP for single incorporation and 30 mg L^−1^ for double incorporation of nhPSer, which is ≈40‐fold higher than the achieved yield by adding 2 mM nhPSer. The drastic increase in protein yield emphasizes the strong influence of charges on the cellular uptake of ncAAs and underlines the potency of in vivo biosynthesis. Finally, sfGFP alloproteins with incorporated PSer and nhPSer ncAAs were evaluated for resistance to enzyme‐catalyzed hydrolysis by incubation with λ‐phosphatase. This resulted in hydrolyzed protein where PSer was converted to Ser and nonhydrolyzed nhPSer‐containing alloprotein. Among other applications, nhPSer was shown to effectively mimic PSer by priming GSK3β, resulting in the same pattern of polyphosphorylation in the SARS‐CoV‐2 nucleocapsid protein as observed with PSer.

Tyr sulfation is an important post‐translational modification of proteins, involved in processes such as chemotaxis and plant immunity.^[^ ^42^ ^]^ Xiao and colleagues^[^ ^43^ ^]^ therefore set out to engineer autonomous cells capable of producing sulfotyrosine (STyr, **82**) containing alloproteins. To identify a suitable enzyme for Tyr sulfation, different sulfotransferase variants that utilize 3′‐phosphoadenosine‐5′‐phosphosulfate (PAPS) as a cofactor were screened. Efficient STyr biosynthesis was measured through STyr incorporation into sfGFP_151TAG via the previously published *M. jannaschii*‐derived OTS STyrRS.^[^ ^44^ ^]^ For the screening, a three‐plasmid system and *E. coli* BL21(DE3) as the host organism were used. As the rational decision for a suitable sulfotransferase did not yield sfGFP_STyr, 27 sequences derived from sequence similarity networks were evaluated. The authors found the sulfotransferase A0A091VQH7 from *Nipponia nippon*, which was named *Nn*SULT1C1, to result in the highest level of sfGFP fluorescence. To improve the biosynthesis of STyr, *cysH*, which degrades the cofactor PAPS to 3′‐phosphoadenosine 5′‐phosphate, was knocked out. They also found that the expression of the *cysDNCQ* genes led to higher levels of sfGFP_STyr expression. This is because these genes encode a system needed for efficient PAPS recycling. The application of this system resulted in a 28‐fold higher concentration of intracellular STyr compared to exogenous feeding of STyr at 1 mM and more than twofold compared to supplementation with 27 mM STyr. The implemented STyr biosynthetic pathway also resulted in an almost fourfold higher isolated yield of sfGFP (5.67 mg L^−1^) in contrast to 1 mM exogenously fed STyr (sfGFP 1.5 mg L^−1^). The application of the *Nn*SULT1C1 sulfotransferase system in HEK293T cells with the *Ec*TyrRS/tRNA OTS^[^ ^45^ ^]^ and EGFP_39TAG reporter leads to higher EGFP expression levels in HEK293T‐*Nn*SULT1C1 cells than in HEK293T cells fed with 3 mM STyr. By incorporating STyr into the thrombin inhibitors madanin‐1 and chimadin at two different sites, the cited study could prove a strong increase in thrombin inhibition efficiency, highlighting the utility of tyrosine sulfation in putative applications.

**Figure 3:**
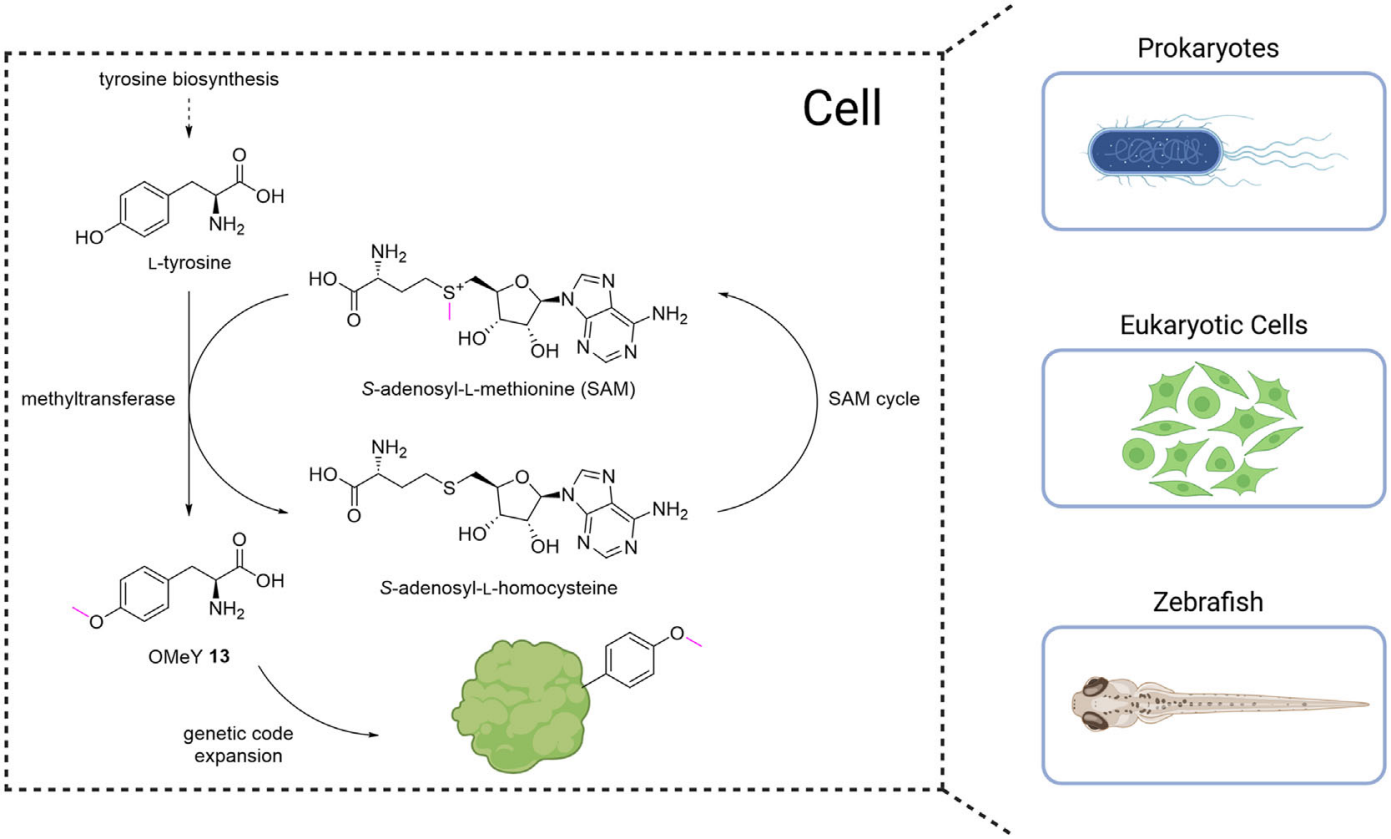
Illustration of the in vivo synthesis of OMeY **13** via the methyltransferase MfnG from *Streptomyces drozdowiczii*. The cofactor SAM, which serves as a methyl donor (shown in purple), was recycled using the endogenous SAM recycling system. Autonomous prokaryotic cells, eukaryotic cell lines, and zebrafish that biosynthesize and incorporate OMeY were demonstrated. The figure was adapted from Xiao and colleagues with permission from ref. [45] (Copyright Wiley, 2022) and partially created in https://BioRender.com.

In another exciting approach which uses a cofactor‐dependent enzyme, Xiao and colleagues^[^ ^46^ ^]^ showed that they could biosynthesize and incorporate *O*‐methyltyrosine (OMeY, **13**) in HEK cells, zebrafish, and the more commonly used *E. coli* (**Figure** **3**). Five different *O*‐methyltransferases, which use tyrosine and *S*‐adenosyl‐l‐methionine (SAM) as metabolic methyl donors, were validated for OMeY biosynthesis. For the direct evaluation of combined ncAA biosynthesis and incorporation, a *M. jannaschii*‐derived OTS encoded on the plasmid pUltra‐polyRS for site‐specific incorporation was employed. Moreover, two additional plasmids were introduced, carrying sfGFP_134TAG as a reporter protein and the *O*‐methyl‐transferase. Inducing the methyltransferase MfnG from *Streptomyces drozdowiczii* ^[^ ^47^ ^]^ in *E. coli* BL21(DE3) with sfGFP_134TAG as readout resulted in a tenfold higher fluorescence than the uninduced control.

Moreover, it also exceeded the fluorescence levels of all other transferases evaluated. Compared to exogenously fed OMeY (1 mM), in situ biosynthesis achieved 2–2.5‐fold higher fluorescence levels by using the metabolic conversion of tyrosine and SAM to OMeY. Structural analysis of MfnG revealed a hydrophobic pocket that accommodates the aromatic core of Tyr. Hence, it was hypothesized that MfnG was likely to tolerate other benzylic substrates as well, potentially giving rise to novel ncAAs in future research. To incorporate OMeY into EGFP in mammalian HEK293T cells, two orthogonal tRNA copies derived from *Bacillus stearothermophilus* and *E. coli* tyrosyl tRNA were used, as well as the *E. coli* tyrosyl tRNA synthetase. MfnG was again used for biosynthesis. Compared to exogenously fed OMeY (1 mM), HEK293T_MfnG cells showed 2.4 times higher fluorescence, and the yield of purified EGFP was more than three times higher than with exogenous supplementation of OMeY. In addition, when a construct similar to the one used in HEK cells was introduced into the vertebrate zebrafish, higher fluorescence levels were detected in the zebrafish with biosynthesized OMeY than in the control group with exogenous supplementation.

**Scheme 6:**
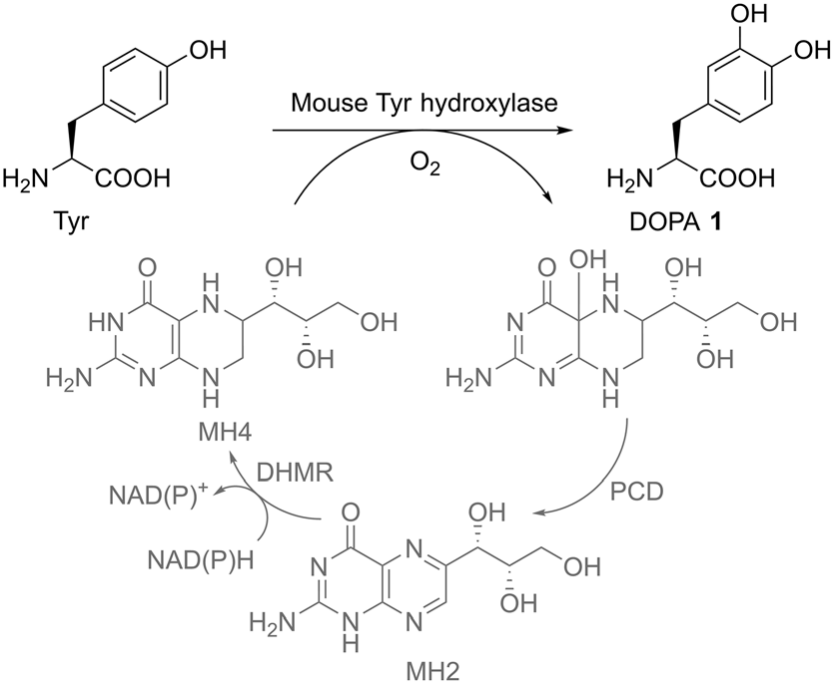
Biotransformation of Tyr to DOPA (**1**), which is catalyzed by mouse tyrosine hydroxylase and requires molecular oxygen and the cofactor MH4. The schematic also illustrates the MH4 recycling pathway (marked in gray). PCD converts the intermediate to dihydromonapterin (MH2), which is then reduced back to MH4 by DHMR using NAD(P)H as an electron donor.

While working on the engineering of the DOPA OTS, Thyer et al.^[^ ^48^ ^]^ also developed a fluorescent biosensor to monitor DOPA incorporation and engineered autonomous DOPA (**1**) cells. Misincorporation of Tyr is a common challenge for *Mj*TyrRS DOPA OTSs. Therefore, a system that could discriminate between DOPA and Tyr incorporation was sought. In a previous report, DOPA incorporation into GFP was shown to result in nonfluorescent cells.^[^ ^49^ ^]^ Therefore, the researchers replaced eight Tyr codons in sfGFP with the amber codon TAG. Subsequently, sfGFP was genetically fused to chloramphenicol acetyltransferase (CAT) (with four in‐frame amber codons), resulting in nonfluorescent, but chloramphenicol‐resistant cells when DOPA was incorporated. On the other hand, the misincorporation of Tyr should result in fluorescent and chloramphenicol‐resistant cells. With this system in hand, five positions of *Mj*TyrRS were randomized, resulting in several colonies with high sequence similarity and one clone with distinct mutations, which proved to be the only clone able to incorporate DOPA. To overcome the strict qualitative readout of the sfGFP‐CAT screening system, a direct fluorescent biosensor of DOPA incorporation was sought. Inspired by a previous publication,^[^ ^50^ ^]^ the researchers searched for a GFP mutation that caused a redshift when DOPA was incorporated. The mutation S205C was identified, which resulted in excitation at 535 nm and emission at 585 nm when DOPA was incorporated at position 66. However, this system did not identify another useful mutation in the *Mj*TyrRS DOPA OTS. To establish the biosynthesis, the researchers explored *E. coli* 4‐hydroxyphenylacetate 3‐monooxygenase, which hydroxylates Tyr to DOPA, and HpaC, which recycles the cofactor FAD. The authors of the study used *E. coli* B95.ΔA cells in a three‐plasmid system. After expression, the characteristic redshift of GFP described above was observed, thus demonstrating combined biosynthesis and DOPA incorporation. In a later study, Xiao and colleagues^[^ ^51^ ^]^ demonstrated that they gained higher levels of alloproteins by biosynthesizing and incorporating DOPA than what could be achieved by exogenously feeding DOPA. For the incorporation of DOPA, two known OTSs were first evaluated,^[^ ^13^, ^23^ ^]^ as misincorporation of Tyr is a known challenge occurring for the *Mj*TyrRs DOPA OTS.^[^ ^48^ ^]^ It was found that using chimeric PheRS/tRNA^[23a]^ from human mitochondria resulted in sfGFP without misincorporation, and the highest fluorescence level was achieved when 9 mM DOPA was fed. To establish the biosynthesis, several hydroxylases from different species were screened to identify a hydroxylase that efficiently oxidizes Tyr to DOPA. In addition, a tetrahydromonapterin (MH4) recycling pathway was established, as all hydroxylase enzymes evaluated were MH4‐dependent. This plasmid‐based recycling pathway consisted of the dihydromonapterin reductase (DHMR) from *E. coli*, which converts dihydroxymonapterin to MH4, and pterin‐4α‐carbinolamine dehydratase (PCD) from *Pseudomonas aeruginosa*, which regenerates dihydroxymonapterin (**Scheme** **6**). To evaluate which hydroxylase is most suitable for the oxidation of Tyr, a two‐plasmid system was used in *E. coli* BL21(DE3), with one plasmid containing the OTS for incorporation and the other plasmid containing sfGFP_134TAG, the MH4 recycling system, and the hydroxylase of interest. In addition, vitamin C was added during expression to prevent DOPA oxidation. The study found that DOPA biosynthesis based on mouse tyrosine hydroxylase enabled the isolation of sfGFP_DOPA in a yield of 3.1 mg L^−1^, which was about 20% higher than the control in which 9 mM DOPA was fed (2.5 mg L^−1^ sfGFP_DOPA). Two bioorthogonal reactions were performed after the incorporation of DOPA into the single‐chain fragment of the HER2‐targeting trastuzumab antibody (anti‐HER2) to demonstrate the utility of DOPA incorporation for post‐translational protein modification. Highlighting the practical benefit of DOPA incorporation into proteins, anti‐HER2_DOPA was reacted with *p*AF in an oxidative coupling reaction. Further, site‐specific labeling of the antibody was achieved via SPOCQ reaction with a strained cycloalkyne or oxidative coupling with a fluorescent aniline‐dimethylaminophthalimide probe.^[^ ^51^ ^]^

Looking at a different enzyme that uses the MH4 recycling pathway, an autonomous organism that uses 5‐hydroxytryptophan (5OHW, **83**) as a 21st amino acid was engineered. With this autonomous organism, Xiao and colleagues^[^ ^52^ ^]^ demonstrated that the levels of sfGFP_5OHW protein can serve as a monitor of oxidative stress in bacterial cells. For the biosynthesis, different phenylalanine 4‐hydroxylases that use MH4 as a cofactor were explored. They evaluated four hydroxylases from different species together with the previously introduced MH4 recycling pathway (cf. DOPA biosynthesis). The tryptophanyl‐tRNA synthetase (*Sc*TrpRS)/tRNA^Trp^ OTS from *Saccharomyces cerevisiae* ^[^ ^53^ ^]^ was used for incorporation into the reporter protein sfGFP, which is known to efficiently incorporate 5OHW at low levels of 0.1 mM. Expression was carried out in *E coli* BL21(DE3) using a dual plasmid system, one plasmid containing sfGFP, the hydroxylase, and the MH4 recycling pathway, the other plasmid the OTS on a pUltra vector.^[^ ^54^ ^]^ The highest level of sfGFP fluorescence, and thus, the highest rate of 5OHW biosynthesis and incorporation was observed with the phenylalanine 4‐hydroxylase of *Xanthomonas campestris*. An internal concentration of 60 μM was obtained for biosynthesis, resulting in a final concentration of 10.5 mg L^−1^ isolated sfGFP compared to 12.5 mg L^−1^ sfGFP when fed with 1 mM 5OHW (907.5 μM cellular level of 5OHW). Incorporation into the anti‐HER2‐scFV antibody and coupling to coumarin diazonium via a chemoselective azo‐coupling reaction demonstrated site‐specific fluorophore labeling with over 90% labeled protein. To use these 5OHW‐producing autonomous cells as an indicator of oxidative stress, the bacteria were incubated with hydrogen peroxide as a source of oxidative stress. Lower levels of sfGFP_5OHW produced corresponded to higher levels of hydrogen peroxide, which was explained by the lower availability of other cofactors for biosynthesis.

## 3 Current Opinion and Discussion

When discussing the incorporation of ncAAs into proteins, it is important to consider when the benefit is worth the effort. However, this is not an easy question to answer. To narrow the scope of the discussion, this section focuses solely on aspects of biosynthesis and direct incorporation, rather than the functional benefits of incorporated ncAAs themselves. This has been emphasized in prior reviews[^8^, ^55^] and is evident from the examples of potential medical or industrial applications.

In general, the associated costs when using ncAAs must be carefully considered, since most ncAAs are expensive to synthesize or acquire.^[^ ^56^ ^]^ The estimated cost of an industrial commercial‐scale fermentation of alloproteins, dependent on ncAA supplementation, is in the region of several million euros.^[^ ^36^, ^57^ ^]^ In certain cases, the economic challenge is compounded by the need for high extracellular ncAA concentrations in order to create an effective concentration gradient for cellular uptake.^[^ ^43^ ^]^ These requirements make large‐scale ncAA supplementation for alloprotein fermentation economically impractical. Using autonomous cells can reduce process costs as no ncAA supplementation is necessary and can therefore be considered as the gold standard for the use of ncAAs. However, supplying precursors remains a compelling strategy as it offers the possibility of biosynthesizing and incorporating a large variety of ncAAs^[^ ^24^ ^]^ and also significantly reduces the cost factor.^[^ ^11^ ^]^

In terms of process, it should be considered whether higher levels of the product, i.e., the target protein, can be achieved by combining biosynthesis and incorporation. Several examples suggest that the combination of biosynthesis and incorporation leads to higher levels of the target protein.^[^ ^11^, ^19^, ^46^, ^51^ ^]^ However, in some examples, the opposite was found.^[^ ^33^, ^52^ ^]^ These mixed outcomes suggest no universal trend. One explanation could be that the interaction between biosynthesis and incorporation is essential. This means that for an OTS that requires elevated levels of ncAA for incorporation, efficient biosynthesis and metabolic flux are necessary to reach these intracellular levels. Conversely, an efficient OTS does not require biosynthesis to reach high intracellular ncAA concentrations. It could be argued that this is even detrimental, since high concentrations of ncAAs can impair cell growth and have cytotoxic effects, and their biosynthesis could represent a metabolic burden for the host organism.^[^ ^58^ ^]^ For example, it was shown that in one case of SPI incorporation, the levels of biosynthesized ncAA were too low to incorporate the ncAA at multiple sites in the protein.^[^ ^16^ ^]^ The importance of biosynthesis was also demonstrated for an approach using two different ncAAs, as the resulting protein yield was significantly dependent on the efficiency of biosynthesis.^[^ ^29^ ^]^ In another case, the OTS was shown to be the bottleneck, and engineering the OTS almost doubled the amount of target protein with incorporated ncAA.^[^ ^24^ ^]^

Strain engineering can play a role in multiple ways, when engineering (semi) autonomous cells, for example, to reduce misincorporation with canonical amino acids.^[^ ^16^, ^38^ ^]^ In addition, targeted strain engineering can increase the availability of metabolic precursors required for ncAA biosynthesis.^[^ ^18^ ^]^ Furthermore, strains optimized for the usage of alternative stop codons and release factor knock‐out have shown increased protein yields in some cases,^[^ ^30^ ^]^ though not universally.^[^ ^21^ ^]^ Another important way in which strains can be engineered is through the stable integration of OTSs or biosynthetic pathways in the genome. This can reduce the metabolic burden, replication control conflicts, and vector instability compared to the vector‐based approaches described in this review.^[^ ^59^ ^]^ Further optimization of genomic OTS integration is possible via adaptive laboratory evolution or genomic integration experiments designed to stabilize the expanded genetic code under selection pressure, for instance.[^59^, ^60^]

Finally, when deciding whether biosynthesis and uptake are essential, the ncAA itself must also be considered, as the membrane permeability of the ncAA might be low. This is especially evident for charged ncAAs, often showcasing poor cellular uptake.^[^ ^40^, ^43^, ^61^ ^]^ It has also been described that very hydrophobic, hydrophilic, or bulky ncAAs have poor membrane permeability.^[^ ^21^, ^51^, ^62^ ^]^ This means that very high levels of supplemented ncAA are required in these cases when fed exogenously, which, on the one hand, increases the cost of the process. On the other hand, the potentially necessary intracellular levels cannot be achieved without biosynthesis, demonstrating the necessity of (semi)autonomous cells.

## 4 Conclusions and Outlook

In conclusion, semi‐autonomous and autonomous cell systems provide powerful tools to reduce production costs, bypass the challenge of membrane permeability, and enhance alloprotein yields. A summary of the approaches discussed in this review is provided in Table S1, Supporting Information.

Further research should focus on optimizing the complex biosynthesis and incorporation interplay. Furthermore, identifying and implementing additional biosynthetic pathways and enhancing the translation machinery will be of importance. This could be driven by OMICS data, enabling rational and systemic interventions, as well as artificial intelligence‐driven pathway evolution and predictive modeling.^[^ ^43^, ^63^ ^]^ Moreover, reassigned sense codons via whole‐genome engineering or synthetic cells have the potential to circumvent current limitations of SPI and SCS, enabling more efficient alloprotein production.^[^ ^64^ ^]^

## Conflict of Interest

The authors declare no conflict of interest.

## Author Contributions

**Jan Hendrik Illies** and **Ivana Drienovská** jointly conceived the scope and structure of this review. **Jan Hendrik Illies** drafted the manuscript, led subsequent revisions and edits, and managed responses to reviewers. **Tim Moritz Weber** contributed to the scientific development of the manuscript and edited the original draft. **Ivana Drienovská** supervised the project, contributed to its conceptual development and edited the original draft.

## Supporting information

Supplementary Material
