# Supplementary material for: In Vivo Biosynthesis and Direct Incorporation of Noncanonical Amino Acids into Proteins
Source: Chembiochem. 2025 Sep 19;26(22):e202500282. doi: 10.1002/cbic.202500282 (PMC12631008; doi:10.1002/cbic.202500282)
Supplement: Supplementary file 1 — Supplementary Material [file CBIC-26-e202500282-s001.pdf]

WILEY-VCH

In Vivo Biosynthesis and Direct Incorporation of Noncanonical Amino Acids into Proteins

Jan Hendrik Illies[a,b], Tim Moritz Weber[a,b], and Ivana Drienovská*[a,b]

Supporting Information

WILEY • VCH

[a] J. H. Illies, T. M. Weber, Dr. I. Drienovská*
Amsterdam Institute of Molecular and Life Sciences (AIMMS)
Vrije Universiteit Amsterdam
De Boelelaan 1105, 1081HV Amsterdam, The Netherlands
*E-mail: i.drienovska@vu.nl
[b] J. H. Illies, T. M. Weber, Dr. I. Drienovská*
Department of Chemistry and Pharmaceutical Sciences
Vrije Universiteit Amsterdam
De Boelelaan 1105, 1081HV Amsterdam, The Netherlands

Table S1: Summary table compiling the (semi-) autonomous cell systems described in the main manuscript. The table includes columns for the name and abbreviation of the ncAA (n.s.: not specified), the corresponding number in the manuscript, the supplemented precursor(s) (not applicable (n.a.) for autonomous cell systems), the enzymes involved in ncAA biosynthesis, the incorporation method, the target protein, and the reference. The approaches are highlighted in color depending on the type of incorporation: *Mj*TyrRS is blue, chPheRS is orange, PylRS is green and *Sc*TrpRS is purple. The SPI approaches remain black.

ncAA	Abbreviation	Number in manuscript	Supplemented precursor (n.a. for autonomous cells)	Enzyme(s) involved	Incorporation method	Protein of incorporation	Reference
L-3,4-dihydroxy-phenylalanine	DOPA	**1**	catechol	tyrosine phenol lyase	SCS (*Mj*TyrRS)	emerald GFP	[1]
		**1**	n.a.	4-hydroxyphenylacetate 3-mono-oxygenase / Mouse Tyr hydroxylase	SCS (*Mj*TyrRS) / SCS (chPheRS)	sfGFP-CAT / sfGFP and anti-HER2	[2]
2-fluorotyrosine	2-FY	**2**	2-fluorophenol	tyrosine phenol lyase	SPI (Tyr auxotrophic strain)	ω transaminase and alanine dehydrogenase	[3]
2,3-difluorotyrosine	2,3-FY	**3**	2,3-difluorophenol	tyrosine phenol Lyase	SPI (Tyr auxotrophic strain)	ω transaminase and alanine dehydrogenase	[3]
3-fluorotyrosine	3-FY	**4**	3-fluorophenol	tyrosine phenol lyase	SPI (Tyr auxotrophic strain)	ω transaminase and alanine dehydrogenase	[3]
3,5-difluorotyrosine	3,5-FY	**5**	3,5-difluorophenol	tyrosine phenol lyase	SPI (Tyr auxotrophic strain)	ω transaminase and alanine dehydrogenase	[3]
S-allyl-L-cysteine	SAC	**6**	allyl-mercaptan	*O*-acetyl-serine sulfhydrylase isoenzymes	SCS (*Mm*PylRS)	EGFP	[4]
S-allyl-L-homocysteine	SAHC	**7**	allyl mercaptan	homoserine acetyltransferase + *O*-acetyl homoserine sulfhydrylase	SPI (Met auxotrophic strain)	GFP	[5]
L-azidohomoalanine	Aha	**8**	*O*-acetyl-L-homoserine, sodium azide	homoserine acetyltransferase + *O*-acetyl homoserine sulfhydrylase	SPI (Met auxotrophic strain)	cysteine-free ψB*	[6]
p-acetyl-L-phenylalanine	*p*AcF	**9**	corresponding keto acid	glutamine:phenylpyruvate aminotransferase	SCS (*Mj*TyrRS)	GFP	[7]
p-azido-L-phenylalanine	*p*AzF	**10**	corresponding keto acid	glutamine:phenylpyruvate aminotransferase	SCS (*Mj*TyrRS)	GFP	[7]
p-phenyl-L-phenylalanine	*p*PhF	**11**	corresponding keto acid	glutamine:phenylpyruvate aminotransferase	SCS (*Mj*TyrRS)	GFP	[7]

WILEY-VCH

ncAA	Abbreviation	Number in manuscript	Supplemented precursor (n.a. for autonomous cells)	Enzyme(s) involved	Incorporation method	Protein of incorporation	Reference
p-cyano-L-phenylalanine	*p*CNF	**12**	corresponding keto acid	glutamine:phenylpyruvate aminotransferase	SCS (*Mj*TyrRS)	GFP	[7]
L-4-methoxy-phenylalanine	OMeY	**13**	*p*-methoxyphenylpyruvic acid	GlnAT/AspAT	SCS (*Mm*PylRS)	EGFP	[8]
		**13**	n.a.	MfnG	SCS (*Mj*TyrRS)	sfGFP, EGFP	[9]
ncAAs **14-21**	n.s.	**14-21**	phenylpyruvic acid variants	GlnAT/AspAT	SCS (*Mm*PylRS)	EGFP	[8]
6-chloro-tryptophan	6ClW	**22**	NaCl	SttH halogenase	SCS (chPheRS)	sfGFP, nano-luciferase, and anti-HER2	[10]
6-bromo-tryptophan	6BrW	**23**	NaBr	SttH halogenase	SCS (chPheRS)	sfGFP, nano-luciferase, and anti-HER2	[10]
7-chloro-tryptophan	7ClW	**24**	NaCl	RebH halogenase	SCS (chPheRS)	sfGFP, nano-luciferase, and anti-HER2	[10]
7-bromo-tryptophan	7BrW	**25**	NaBr	RebH halogenase	SCS (chPheRS)	sfGFP, nano-luciferase, and anti-HER2	[10]
S-phenyl-L-cysteine	*p*AzPhC	**26**	*S*-(4-azidophenyl)-L-cysteine	CysM + *Nt*Sat4 and *At*Sat3	SCS (*Mj*TyrRS)	sfGFP, anti-HER2	[11]
ncAAs **27-73**	n.s.	**27-73**	thiol nucleophile variants	CysM + *Nt*Sat4 and *At*Sat3	SCS (*Mj*TyrRS)	sfGFP, anti-HER2	[11]
desmethylpyrrolysine	dmPyl	**74**	D-ornithine	PylC + PylD	SCS (PylRS)	human fatty acid synthase, mouse tumor necrosis factor alpha, FK506 binding protein 1A, and mouse epidermal growth factor, human retinal binding protein 4, and mouse erythropoietin	[12]
3*S*-ethynylpyrrolysine	ePyl	**75**	3*S*-ethynyl-D-ornithine	PylC + PylD	SCS (PylRS)	human carbonic anhydrase 2	[12b]
D-Cys-ε-Lys	n.s.	**76**	D-Cys	PylC mutant	SCS (PylRS)	mCherry, p16p	[13]
p-aminophenylalanine	*p*AF	**77**	n.a.	PapA + PapB + PapC + *E. coli* aminotransferase	SCS (*Mj*TyrRS)	myoglobin, anti-HER2	[14]

ncAA	Abbreviation	Number in manuscript	Supplemented precursor (n.a. for autonomous cells)	Enzyme(s) involved	Incorporation method	Protein of incorporation	Reference
p-nitrophenylalanine	*p*NF	**78**	n.a.	PapA + PapB + PapC + *E. coli* aminotransferase + NO16	SCS (*Mj*TyrRS)	chimeric ubiquitin-sfGFP	[15]
norleucine	Nle	**79**	n.a.	LeuABCD	SPI (Val, Ile, Leu, Met auxotrophic strain)	OYE and TTL	[16]
O-phospho-L-threonine	PThr	**80**	n.a.	PduX	SCS (*Mj* and *M. maripaludis*)	GFP, cyclin-dependent kinase 2	[17]
non-hydrolysable PSer	nhPSer	**81**	n.a.	FrbD, FrbC, FrbA, and FrbB/FrbE + unknown *E. coli* transaminase	SCS (*Mj* and *M. maripaludis*)	sfGFP, SARS-CoV-2 nucleocapsid protein	[18]
sulfotyrosine	STyr	**82**	n.a.	*Nn*SULT1C1	SCS (*Mj* and *Ec*TyrRS)	sfGFP, EGFP	[19]
5-hydroxytryptophan	5OHW	**83**	n.a.	phenylalanine 4-hydroxylase	SCS (*Sc*TrpRS)	sfGFP, anti-HER2-scFV	[20]

WILEY-VCH

References

[1] S. Kim, B. H. Sung, S. C. Kim, H. S. Lee, *Chem. Commun.* **2018**, *54*, 3002-3005.

[2] a) R. Thyer, S. D'Oelsnitz, M. S. Blevins, D. R. Klein, J. S. Brodbelt, A. D. Ellington, *Angew. Chem.* **2021**, *133*, 14937-14942; *Angew. Chem. Int. Ed.* **2021**, *60*, 14811-14816; b) Y. D. Chen, A. Loredo, A. N. Chung, M. X. Zhang, R. Liu, H. Xiao, *J. Mol. Biol.* **2022**, *434*, 167412.

[3] Y. Won, H. Jeon, A. D. Pagar, M. D. Patil, S. P. Nadarajan, D. T. Flood, P. E. Dawson, H. Yun, *Chem. Commun.* **2019**, *55*, 15133-15136.

[4] M. P. Exner, T. Kuenzl, T. M. T. To, Z. Ouyang, S. Schwagerus, M. G. Hoesl, C. P. R. Hackenberger, M.C. Lensen, S. Panke, N. Budisa, *ChemBioChem* **2017**, *18*, 85-90.

[5] S. Nojoumi, Y. Ma, S. Schwagerus, C. P. R. Hackenberger, N. Budisa, *Int. J. Mol. Sci.* **2019**, *20*, 2299.

[6] Y. Ma, H. Biava, R. Contestabile, N. Budisa, M. L. di Salvo, *Molecules* **2014**, *19*, 1004-1022.

[7] J. Jung, Y. Lee, H. Park, H. Cha, W. Ko, K. Sachin, D. W. Kim, D. Y. Chi, H. S. Lee, *Chem. Sci.* **2014**, *5*, 1881-1885.

[8] L. Liu, B. Wang, S. Li, F. Xu, Q. He, C. Pan, X. Gao, W. Yao, X. Song, *Biomolecules* **2021**, *11*, 1358.

[9] K.-L. Wu, J. A. Moore, M. D. Miller, Y. Chen, C. Lee, W. Xu, Z. Peng, Q. Duan, G. N. Phillips Jr, R. A. Uribe, H. Xiao, *Protein Sci.* **2022**, *31*, e4443.

[10] Y. Guo, L. Cheng, Y. Hu, M. Zhang, R. Liu, Y. Wang, S. Jiang, H. Xiao, *ChemBioChem* **2024**, *25*, e202400366.

[11] a) Y. Wang, X. Chen, W. Cai, L. Tan, Y. Yu, B. Han, Y. Li, Y. Xie, Y. Su, X. Luo, T. Liu, *Angew. Chem. Int. Ed.* **2021**, *60*, 10040-10048; *Angew. Chem.* **2021**, *133*, 10128-10136.

[12] a) W. J. Ou, T. Uno, H. P. Chiu, J. Grünewald, S. E. Cellitti, T. Crossgrove, X. S. Hao, Q. Fan, L. L. Quinn, P. Patterson, L. Okach, D. H. Jones, S. A. Lesley, A. Brock, B. H. Geierstanger, *Proc. Natl. Acad. Sci. U.S.A.* **2011**, *108*, 10437-10442; b) M. Ehrlich, M. J. Gattner, B. Viverge, J. Bretzler, D. Eisen, M. Stadlmeier, M. Vrabel, T. Carell, *Chem. Eur. J.* **2015**, *21*, 7701-7704.

[13] J. Tai, L. Wang, W. S. Chan, J. Cheng, Y. H. Chan, M. M. Lee, M. K. Chan, *J. Am. Chem. Soc.* **2023**, *145*, 10249-10258.

[14] a) R. A. Mehl, J. C. Anderson, S. W. Santoro, L. Wang, A. B. Martin, D. S. King, D. M. Horn, P. G. Schultz, *J. Am. Chem. Soc.* **2003**, *125*, 935-939; b) Y. Chen, A. Loredo, A. Gordon, J. Tang, C. Yu, J. Ordoneza, H. Xiao, *Chem. Commun.* **2018**, *54*, 7187-7190.

[15] N. D. Butler, S. Sen, L. B. Brown, M. Lin, A. M. Kunjapur, *Nat. Chem. Biol.* **2023**, *19*, 911-920.

[16] N. Anderhuber, P. Fladischer, M. Gruber-Khadjawi, J. Mairhofer, G. Striedner, B. Wiltschi, *J. Biotechnol.* **2016**, *235*, 100-111.

[17] M. S. Zhang, S. F. Brunner, N. Huguenin-Dezot, A. D. Liang, W. H. Schmied, D. T. Rogerson, J. W. Chin, *Nat. Methods* **2017**, *14*, 729-736.

[18] P. Zhu, S. Stanisheuski, R. Franklin, A. Vogel, C. H. Vesely, P. Reardon, N. N. Sluchanko, J. S. Beckman, P. A. Karplus, R. A. Mehl, R. B. Cooley, *ACS Cent. Sci.* **2023**, *9*, 816-835.

[19] Y. Chen, S. Jin, M. Zhang, Y. Hu, K. L. Wu, A. Chung, S. Wang, Z. Tian, Y. Wang, P. G. Wolynes, H. Xiao, *Nat. Commun.* **2022**, *13*, 5434.

[20] Y. D. Chen, J. Tang, L. S. Wang, Z. R. Tian, A. Cardenas, X. L. Fang, A. Chatterjee, H. Xiao, *Chem* **2020**, *6*, 2717-2727.

WILEY-VCH
